# *mcr-1*-Mediated In Vitro Inhibition of Plasmid Transfer Is Reversed by the Intestinal Environment

**DOI:** 10.3390/antibiotics11070875

**Published:** 2022-06-29

**Authors:** Xiaoman Yang, Rundong Shu, Leqi Hou, Panpan Ren, Xin Lu, Zhi Huang, Zengtao Zhong, Hui Wang

**Affiliations:** 1 Department of Microbiology, College of Life Sciences, Nanjing Agricultural University, 210095 Nanjing, China; d201980533@hust.edu.cn (X.Y.); 2018116064@njau.edu.cn (R.S.); 2020116063@stu.njau.edu.cn (L.H.); 2020116062@stu.njau.edu.cn (P.R.); zhuang@njau.edu.cn (Z.H.); ztzhong@njau.edu.cn (Z.Z.); 2 Department of Biotechnology, College of Life Science and Technology, Huazhong University of Science and Technology, 430074 Wuhan, China; 3 State Key Laboratory for Infectious Disease Prevention and Control, National Institute for Communicable Disease Control and Prevention, Chinese Center for Disease Control and Prevention, 102206 Beijing, China; luxin@icdc.cn

**Keywords:** *mcr-1*, colistin resistance, conjugation, bile salt, *Klebsiella pneumoniae*, *Escherichia coli*

## Abstract

Colistin is regarded as an antibiotic of last resort against multidrug-resistant Gram-negative bacteria, including *Klebsiella pneumoniae* and *Escherichia coli*. Colistin resistance is acquired by microorganisms via chromosome-mediated mutations or plasmid-mediated mobile colistin resistance (*mcr*) gene, in which the transfer of *mcr* is the predominant factor underlying the spread of colistin resistance. However, the factors that are responsible for the spread of the *mcr* gene are still unclear. In this study, we observed that *mcr-1* inhibited the transfer of the pHNSHP45 backbone in liquid mating. Similar inhibitory effect of *mcr-1.6* and chromosomal mutant Δ*mgrB* suggested that colistin resistance, acquired from either plasmid or chromosomal mutation, hindered the transfer of colistin resistance-related plasmid in vitro. Dual plasmid system further proved that co-existing plasmid transfer was reduced too. However, this inhibitory effect was reversed in vivo. Some factors in the gut, including bile salt and anaerobic conditions, could increase the transfer frequency of the *mcr-1*-containing plasmid. Our results demonstrated the potential risk for the spread of colistin resistance in the intestine, provide a scientific basis against the transmission of colistin resistance threat.

## 1. Introduction

Increasing antibiotic resistance in multidrug-resistant (MDR) Gram-negative bacteria poses a severe threat to public health and safety [1,2]. Due to the lack of development of new antimicrobial agents, an old cationic antimicrobial peptide, colistin, has regained the spotlight in the mid-1990s as the last resort against multidrug-resistant *Enterobacteriaceae*, including *Klebsiella pneumoniae* and *Escherichia coli*. Unfortunately, a rapid global resistance towards colistin has subsequently emerged, which markedly reduces the efficiency of colistin-related antibiotics and makes treatment more difficult. 

Colistin resistance is acquired mainly via two methods. One of the mechanisms is chromosome-mediated mutations. The mutation of *pmrAB* [3], *phoPQ* [4], or *mgrB* [5] is reported to interfere with the synthesis of lipopolysaccharide (LPS) by increasing the modification of lipid A. However, the rate of genomic mutation is low (approximately 10^−9^ to 10^−6^) and easy to reverse [6,7]. The other mechanism is the acquisition of the plasmid-mediated mobile colistin resistance (*mcr*) gene, which encodes a phosphoethanolamine transferase resulting in the addition of phosphoethanolamine to lipid A [8]. Compared with genomic mutations, horizontal gene transfer (HGT) with higher frequency is the predominant mode of acquiring colistin resistance among bacterial cells [9,10].

The first report of mobilizable colistin resistance in 2015 was mediated by pHNSHP45, whose strong transfer frequency is up to 10^−1^ under laboratory conditions [8]. The *mcr-1* gene on pHNSHP45 encodes phosphoethanolamine (pEtN) transferase that alters the cell surface charge by catalyzing the addition of a pEtN to lipid A [11]. To date, various *mcr* variants were widely spread over 60 countries [12,13,14] which raises concerns about the advent of the post-antibiotic era.

However, the acquisition of colistin resistance presumably comes with fitness costs. Our previous study indicated that colistin resistance conferred by either chromosomal *mgrB* deletion or *mcr-1* expression on a plasmid renders carbapenem-resistant *K. pneumoniae* more sensitive to phage infection [15]. It implied that the chemical modifications of the lipopolysaccharide molecules of the outer membrane might change some behavior of recipient bacteria. Plasmid HGT is the predominant method to introduce colistin resistance, and bacterial conjugation is the most efficient strategy for HGT [16,17]. In this study, we examined the transfer frequency of an *mcr-1*-related plasmid in vitro and in vivo using various host strains to evaluate the potential effect of *mcr-1* on gene spreading and the related factors in the process.

## 2. Results

### 2.1. Liquid Conjugation was Established for Quantitative Measurement of Plasmid Transfer Rate

**Figure 1:**
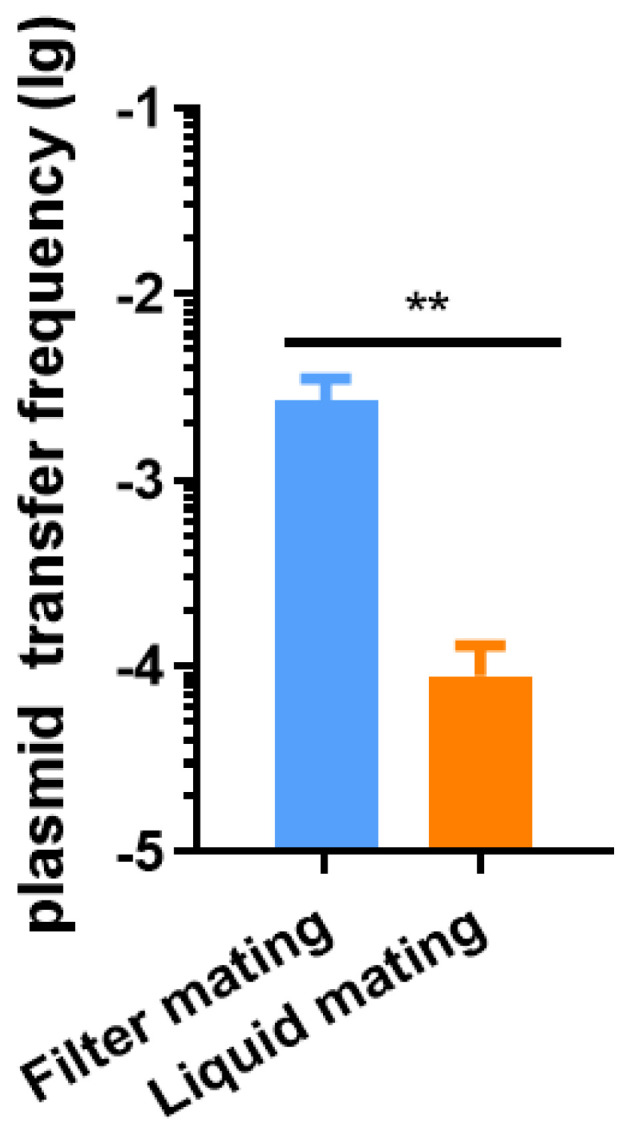
Different conjugation systems for quantitative measurement of plasmid transfer rate. The pHNSHP45 plasmid was transferred from *K. pneumoniae* A2312NM to *K. pneumoniae* D20-2 through a filter or liquid mating. Cultures were incubated till the log phase. Donor and recipient strains were mixed in equal proportions and concentrated 50-fold. The mixture was incubated on a filter or suspended in a liquid medium for 4 h at 37 °C, after which the plasmid transfer frequency was measured. Data are the mean and SD of three independent experiments. Significance was determined using *t*-test; ** *p* < 0.01.

Conjugation is the process of exchanging plasmids between two bacteria. To establish a suitable mating system to observe the variation in the transfer frequency of plasmids, the equal density of donor strain *K. pneumoniae* A2312NM(pHNSHP45) and recipient strain *K. pneumoniae* D20-2 were mixed and incubated on a filter (filter mating) or in a liquid medium (liquid mating). The transfer frequency of the plasmid was determined after conjugation. The pHNSHP45 displayed a high level of transfer (from 10^−3^ to 10^−2^) by filter mating (Figure 1). However, liquid mating exhibited a relatively lower plasmid transfer frequency (from 10^−5^ to 10^−4^), which is suitable to quantify the transfer efficiency variation in this research. Thus, liquid mating was applied to explore the spread of colistin resistance via HGT.

### 2.2. mcr-1 Inhibits pHNSHP45 Transfer through Conjugation

*mcr-1* is carried by the plasmid pHNSHP45, hereafter referred to as the *mcr-1* plasmid. To examine whether the *mcr-1* gene contributes to the transfer of pHNSHP45, we replaced the *mcr-1* gene with a kanamycin-resistant cassette (Km^R^) on pHNSHP45 to obtain the pAC22 plasmid, hereafter named the Δ*mcr-1::Km^R^* plasmid. We also restored the *mcr-1* at an intergenic region on the pAC22 to obtain the pAC23 plasmid, hereafter named the *mcr-1^c^* plasmid. *K. pneumoniae* A2312NM with these different *mcr-1*-related plasmids (*mcr-1*, Δ*mcr-1::Km^R^* and Δ*mcr-1*^c^) were verified using PCR to amplify three evenly distributed genes on pHNSHP45 (*mcr-1*, *parA* and *virB*) and antimicrobial susceptibility testing (Supplementary Figure S1).

**Figure 2:**
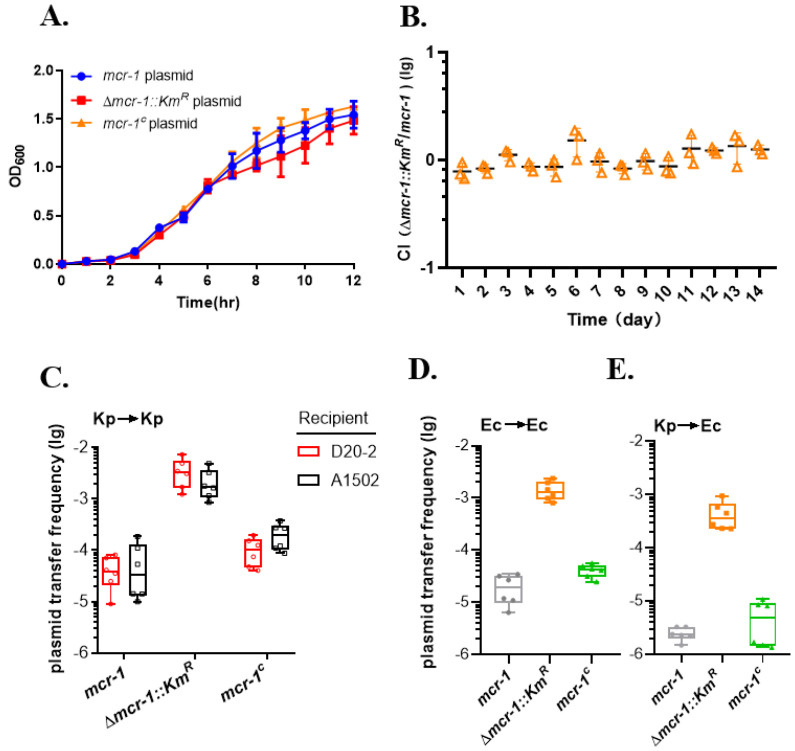
*mcr-1* effect on conjugal transfer of pHNSHP45 plasmid. (**A**) Growth curve of *K. pneumoniae* A2312NM carrying different plasmids. (**B**) Growth competition assay between *K. pneumoniae* A2312NM harboring *mcr-1* plasmid or Δ*mcr-1::Km^R^* plasmid. Samples were transferred to fresh LB medium every day, and CFU was calculated with appropriate antibiotic. Transfer frequency of *mcr-1* plasmid between different strains, including *K. pneumoniae* A2312NM to *K. pneumoniae* D20-2 (red) or A1502 (black) (**C**), *E. coli* MG1655 to *E. coli* Nissle 1917 (**D**), and *K. pneumoniae* A2312NM to *E. coli* MP13 (**E**). An equal volume of donor strain harboring the *mcr-1* plasmid, Δ*mcr-1::Km^R^* plasmid or *mcr-1*^c^ plasmid, and recipient strain were mixed and concentrated 50-fold. Conjugation was performed for 4 h at 37 °C.

*K. pneumoniae* A2312NM harboring a plasmid with or without the *mcr-1* gene exhibited no difference in terms of growth (Figure 2A), suggesting that *mcr-1* expression had no significant effect on the growth of A2312NM. Moreover, no competition was observed between A2312NM strains carrying the *mcr-1* plasmid and the Δ*mcr-1::Km^R^* plasmid when they were cultured together (Figure 2B). The transfer frequency of the *mcr-1* plasmid, Δ*mcr-1::Km^R^* plasmid*,* and *mcr-1^c^* plasmid from *K. pneumoniae* A2312NM to *K. pneumoniae* D20-2 were firstly measured to evaluate the influence of the *mcr-1* gene. The spontaneous mutation rate was approximately 10^−8^ (data not shown), while the conjugation frequency of the *mcr-1* plasmid ranged from 10^−5^ to 10^−4^ (Figure 2C, red). The Δ*mcr-1::Km^R^* plasmid exhibited a transfer frequency of 10^−3^ to 10^−2^, which was approximately 100-fold higher than that of the *mcr-1* plasmid. In addition, the *mcr-1*^c^ plasmid restored the plasmid transfer frequency (Figure 2C, red), suggesting that the *mcr-1* gene had a negative effect on the conjugal transfer of the plasmid backbone. To explore whether this phenotype is strain-specific, we repeated this experiment with *K. pneumoniae* A1502 as the recipient strain. Similar results demonstrated that the plasmid transfer with the *mcr-1* plasmid (the *mcr-1* plasmid ranged from 10^−5^ to 10^−4^) was lower than the plasmid without the *mcr-1* gene (the Δ*mcr-1::Km^R^* plasmid ranged from 10^−3^ to 10^−2^)(Figure 2c, black). Further, the transfer frequency of the *mcr-1^c^* plasmid was reduced from 10^−4^ to 10^−3^. These results suggested that *the mcr-1* gene inhibited the conjugal transfer of its plasmid backbone within *K. pneumoniae*.

We further extend the assay to *E. coli*. The related plasmids were introduced into the conjugal donor strain *E. coli* MG1655. Compared to the transfer frequency of the *mcr-1* plasmid, the deletion of the *mcr-1* gene had an approximately 100-fold increase (Figure 2D), indicating that the *mcr-1* gene inhibited the conjugal transfer rate when the plasmid was transferred from *E. coli* MG1655 to *E. coli* Nissle 1917.

To assess the effect of the *mcr-1* gene on the plasmid transfer between different genera, conjugation was performed between the donor *K. pneumoniae* A2312NM and the recipient *E. coli* MP13. Interestingly, consistent with the aforementioned results, the *mcr-1* plasmid and the *mcr-1^c^* plasmid exhibited a lower transfer frequency than the Δ*mcr-1::Km^R^* plasmid (Figure 2E). Collectively, *mcr-1* inhibited the transfer of the plasmid backbone through conjugation, and this negative effect is common and not limited to any specific bacteria.

### 2.3. Colistin Resistance has Inhibitory Effect on Plasmid Transfer

**Figure 3:**
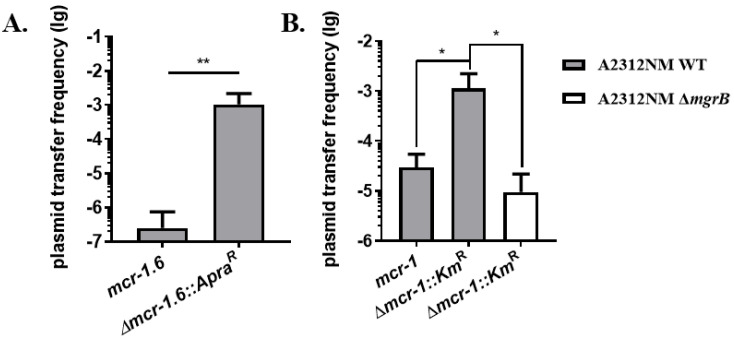
The impact of plasmid or chromosome mediated colistin resistance on plasmid transfer. (**A**) Transfer frequency of *mcr-1*.6 plasmid and Δ*mcr-1*.6*::Apra^R^* plasmid from *K. pneumoniae* A2312NM to *E. coli* MP13. (**B**) *mcr-1*-related plasmid transfer frequency. Donor strains were A2312NM Δ*mgrB* containing Δ*mcr-1::Km^R^* plasmid and A2312NM containing *mcr-1* plasmid or Δ*mcr-1::Km^R^* plasmid. They were conjugated individually with the recipient strain *K. pneumoniae* D20-2. Data are mean and SD of three independent experiments. Significance was determined using *t*-test; * *p* < 0.05, ** *p* < 0.01.

Since the first report of IncI2-type plasmid pHNSHP45, the *mcr-1* gene was found to be carried by diverse plasmid replicon types, such as IncI2, IncHI2, IncP, IncFIP, and IncX4 [18,19,20,21,22]. The previous data implied an inhibitory effect of *mcr-1* on the IncI2 plasmid pHNSHP45. We further repeated it with an IncP Plasmid pMCR1.6 _ P053 [23]. An *mcr-1* variant, named *mcr-1.6*, was carried by pMCR1.6_P053. Compared with *mcr-1*, *mcr-1.6* contains two single-nucleotide polymorphisms that do not impact the activity of phosphoethanolamine transferases. An *mcr-1*.6-deletion plasmid, hereafter named the Δ*mcr-1.6*::*Apra^R^* plasmid, was constructed by replacing the *mcr-1.6* gene fragment with an apramycin-resistant cassette in pMCR1.6_P053. Further, we compared the transfer frequency of the pMCR1.6_P053 plasmid (carrying *mcr-1*.6 gene) and Δ*mcr-1*.*6*::*Apra^R^* from *K. pneumoniae* A2312NM to *E. coli* MP13. The transfer frequency of pMCR1.6_P053 was 2.51 × 10^−7^ (Figure 3A), which was significantly lower than that of the Δ*mcr-1.6*::*Apra^R^* plasmid. It indicated that *mcr-1*.*6* inhibited the conjugal transfer of the plasmid.

Both chromosomal mutations (such as the Δ*mgrB* mutant, which alters the structure of LPS by increasing the modification of Lipid A) and the acquisition of colistin-resistant plasmids can provide colistin resistance to the bacterial cells [24]. To study whether the inhibitory effect is due to the externally derived colistin resistance gene, we tested the Δ*mgrB* mutant of *K. pneumoniae* A2312NM (A2312NM Δ*mgrB*), which possesses chromosome-mediated colistin resistance [15]. Then Δ*mcr-1::Km^R^* plasmid was introduced to A2312NM Δ*mgrB*. Consistent with previous results in Figure 2C, when the conjugation was performed with the same donor strain *K. pneumoniae* A2312NM WT and recipient strain *K. pneumoniae* D20-2, the transfer frequency of the *mcr-1*-containing plasmid (*mcr-1* plasmid) was 100-fold lower than that of the deletion of the *mcr-1* gene plasmid (Δ*mcr-1::Km^R^* plasmid) (Figure 3B). However, the transfer frequency of the Δ*mcr-1::Km^R^* plasmid was 10^−5^ in the A2312NM Δ*mgrB* donor (a chromosomally mediated colistin-resistant strain), which was approximately 100-fold less than in the A2312NM WT donor (a colistin-sensitive strain; Figure 3B). These data indicated that colistin resistance, either from the acquisition of plasmid-mediated *mcr* or chromosomal mutation, can inhibit the transfer of the *mcr-1*-related plasmid.

### 2.4. The Impact of Colistin Resistance on Helper Plasmid Transfer

**Figure 4:**
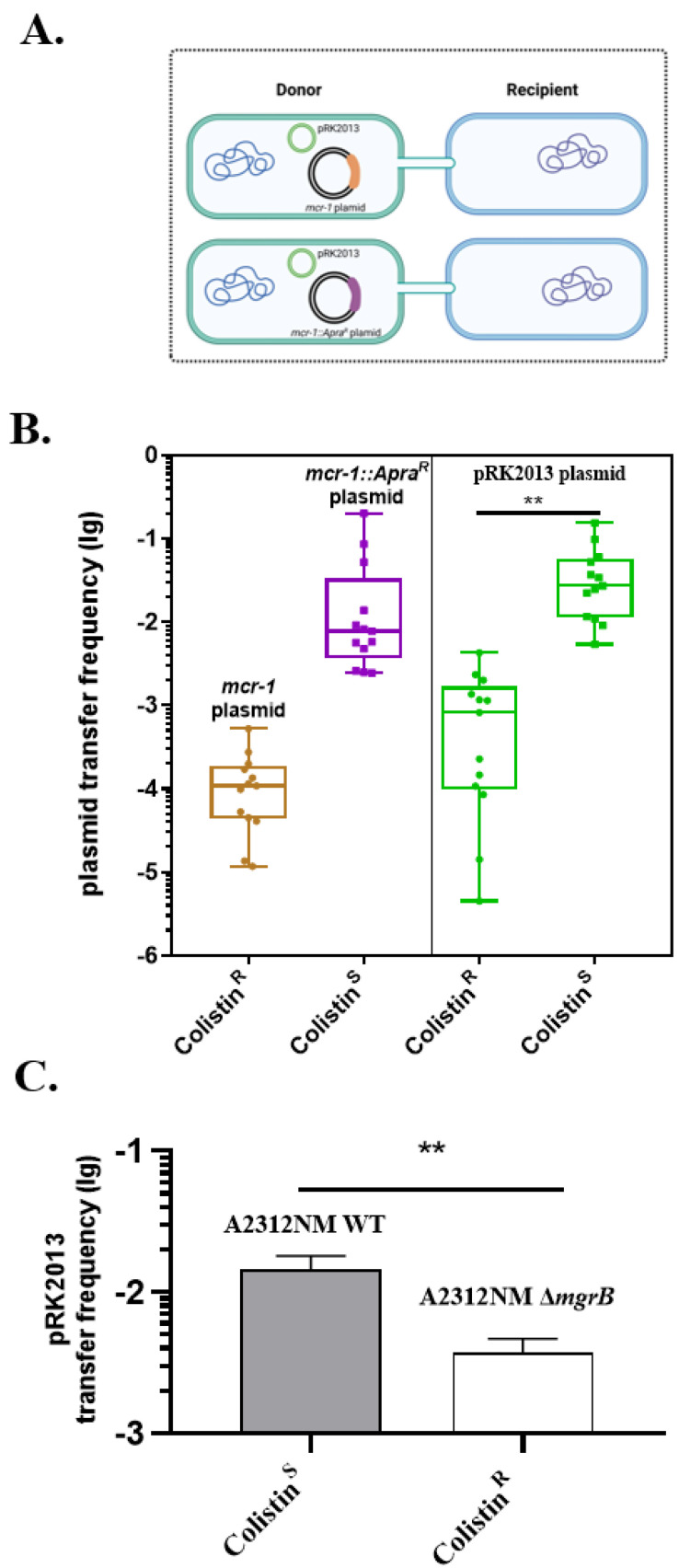
The impact of colistin resistance on the transfer frequency of co-existing plasmid. (**A**) A dual plasmid system was constructed with pRK2013 combined with an *mcr-1* plasmid (orange) or Δ*mcr-1::Apra^R^* plasmid (purple) in the donor strain *K. pneumoniae* A2312NM. (**B**) Conjugation frequency of *mcr-1* or Δ*mcr-1::Apra^R^* plasmid (left) and pRK2013 in the dual plasmid system was measured separately. (**C**) Conjugation frequency of pRK2013 with donor *K. pneumoniae* A2312NM WT or A2312NM Δ*mgrB* and recipient *K. pneumoniae* D20-2. Data are the mean and SD of more than three independent experiments. The significance was determined using *t*-test; ** *p* < 0.01.

As previously shown, the colistin-resistant gene expression on a plasmid had a negative effect on its backbone transfer. Further study was performed to explore the effect of colistin resistance on plasmids that are not related to colistin resistance. We developed a dual plasmid system where the helper plasmid pRK2013 and the *mcr-1*-related plasmids (*mcr-1* plasmid or Δ*mcr-1*::*Apra^R^* plasmid which was also named as pAC24) co-existed in the donor strain *K. pneumoniae* A2312NM (Figure 4A). Liquid mating was performed with the recipient strain *K. pneumoniae* D20-2. As Figure 4B shows, the conjugation frequency of the *mcr-1* plasmid was lower than that of the Δ*mcr-1*::*Apra^R^* plasmid (Figure 4B, left). Meanwhile, the conjugation frequency of pRK2013 was at approximately 10^−3^ when it co-existed with the *mcr-1* plasmid, which was approximately 52 times less than that of pRK2013 co-existed with the Δ*mcr-1*::*Apra^R^* plasmid (Figure 4B, right), the trend is similar to that of the *mcr-1*-related plasmids.

Transfer of pRK2013 under chromosomal-mediated colistin resistance was also tested. A2312NM(pRK2013) (colistin-sensitive) or A2312NM Δ*mgrB*(pRK2013) (colistin-resistant) were set as the donor strain, and *K. pneumoniae* D20-2 acted as the recipient strain. A consistent trend was shown in Figure 4C that the frequency for pRK2013 transfer without colistin resistance was 3.85 folds higher than that with *mgrB*-mutation-mediated colistin resistance. Collectively, these data indicated that the co-existing plasmid transfer was affected by colistin resistance.

### 2.5. mcr-1 Plasmid Transfers In Vivo

**Figure 5:**
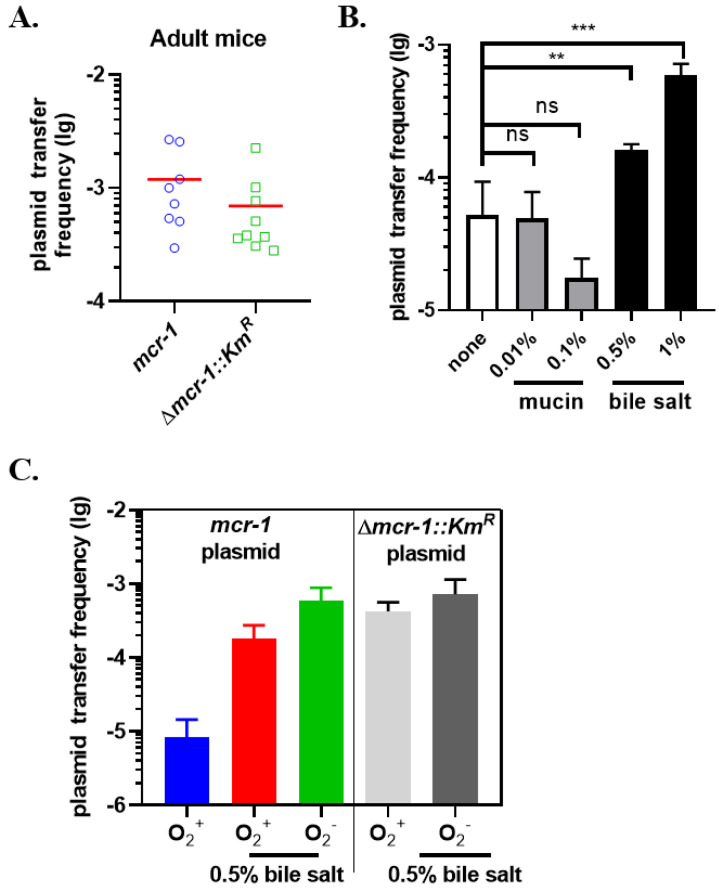
Influence of oxygen and bile salt on plasmid transfer. (**A**) Plasmid transfer in vivo. Five-week-old CD-1 mice were pretreated with streptomycin. Approximately 10^8^ cells of different donor strains, namely, *K. pneumoniae* A2312NM harboring *mcr-1* plasmid, A2312NM containing the Δ*mcr-1::Km^R^* plasmid, were mixed with recipient strain *K. pneumoniae* MP13 separately and immediately administered to each mouse intragastrical. Fecal samples were collected after 3 days, and the transfer frequency was calculated. (**B**) The effect of mucin and bile salt on the transfer of *mcr-1* plasmid. Conjugation was performed with or without additional mucin or bile salt. (**C**) Conjugation was performed with or without bile salt under different oxygen concentrations. Data are the mean and SD of three and more independent experiments. The significance was determined using *t*-test; ns, no significance; ** *p* < 0.01, *** *p* < 0.001.

The intestinal tract is regarded as a ‘melting pot’ for gene exchange which provides several ideal conditions, such as high density and diversity of microbiota, stable temperature, biofilm formation, and so on [25,26,27]. To explore the transfer of *mcr-1* in vivo, we selected the indigenous bacteria *E. coli* MP13 as the recipient strain while *K. pneumoniae* A2312NM carrying the *mcr-1* plasmid or the Δ*mcr-1::Km^R^* plasmid as the donor strain. Adult mice were pretreated with streptomycin to clean the intestinal flora [28,29]. An equal volume of the donor and recipient strain was mixed and immediately administered to the mice intragastrical. Fecal samples were collected after 3 days. The transfer frequency of the *mcr-1* plasmid (1.19 × 10^−3^) was higher than that of the Δ*mcr-1::Km^R^* plasmid (6.93 × 10^−4^), which was opposite to the previous trends in vitro (Figure 5A, Figure 2E), and suggested that colistin resistance support the plasmid transfer in vivo.

The intestine is a complex mini-ecosystem with lots of specific environmental factors which might influence the HGT [30]. Several key factors (mucins, bile salt, and anaerobic conditions) were introduced in the liquid mating system in vitro with the same donor and recipient strains in vivo to test the potential impact. First, we determined the transfer frequency of the *mcr-1* plasmid in the presence of mucin or bile salt. Mucins are the major macromolecular constituent of mucus which act as a barrier in the intestinal epithelium [31,32], and bile salt are an important component of bile that provide not only antibacterial protection in the intestine but also signaling molecules for virulence expression of multiple pathogens [33]. No significant difference was observed in terms of conjugation frequency with an additional 0.01% of mucin in the LB medium, whereas it decreased by 3-fold in the presence of 0.1% mucin (Figure 5B). Interestingly, the transfer frequency of the *mcr-1* plasmid increased by 3–11 fold with bile salt, which was consistent with the results of conjugation in vivo (Figure 2E, Figure 5A,B). This suggested that bile salt facilitates the conjugation-mediated spread of colistin resistance among bacterial cells.

Anaerobic condition is an important signal that is involved in a variety of physiological activities in the gut flora [30]. To evaluate whether oxygen concentration has an impact on bile salt-facilitated plasmid transfer, we performed conjugation under aerobic (shown as “O_2_^+^”) and anaerobic conditions (shown as “O_2_^–^”). The transfer frequency of the *mcr-1* plasmid under aerobic conditions was 10^−5^ (Figure 5C, blue). After the addition of 0.5% bile salt, the frequency notably increased by 21-fold (Figure 5C, red). It kept increasing to approximately 10^−3^ plus anaerobic treatment (Figure 5C, green), suggesting that both bile salt and anaerobic condition play an important role in the process.

Subsequently, we measured the transfer frequency of the Δ*mcr-1::Km^R^* plasmid under O_2_^+^ conditions. The transfer frequency of the Δ*mcr-1::Km^R^* plasmid was 4.21 × 10^−4^, which was 50-fold higher than that of the *mcr-1* plasmid (Figure 5C, blue and gray). It was consistent with previous results that *mcr-1* inhibits the conjugal transfer of the pHNSHP45 plasmid (Figure 2C–E). Conjugation under O_2_^+^ conditions or O_2_^-^ conditions combined with bile salt exhibited similar transfer frequencies around 10^−3^ (Figure 5C, gray and dark gray). We stopped testing more factors because the transfer frequency in this situation is too high to observe additional promotion. In summary, intestinal environmental factors such as bile salt and anaerobic conditions may increase plasmid transfer frequency and thus accelerate the spread of colistin resistance. The intestine is a potential niche for the spread of conjugative plasmids.

## 3. Discussion

HGT via conjugation is considered one of the major contributors to the spread of antibiotic resistance. Many factors are included in affecting the transfer of plasmids.

One of the factors is the nature of the strain. It was reported that the recipient bacteria can influence the yield of transconjugants when the pVCM29188_146 plasmid was transferred to *Salmonella Kentucky* CVM29188, *S. Newport* SL317, and *E. coli* DH10B [34,35]. Moreover, the transfer frequency of RP1/RP4 was 2.05 × 10^−1,^ with *E. coli* HB101 as the donor and *E. coli* X7 as the recipient. The transfer frequency was 2.56 × 10^−2^ when the RP1/RP4 plasmid was transferred within *E. coli* BJ4 itself [36]. Liu reported that the transfer frequency of pHNSHP45 can reach up to 10^−1^ with *E. coli* SHP45 as the donor and *E. coli* C600 as the recipient using the filter mating technique [8]. Our results revealed that pHNSHP45 had a high rate of transfer frequency (10^−2^) in filter mating when it was transferred from *K. pneumoniae* A2312NM to *K. pneumoniae* D20-2 (Figure 1). However, when pHNSHP45 was transferred from *K. pneumoniae* A2312NM to *E. Coli* MP13 instead of *K. pneumoniae* D20-2, the transfer frequency was decreased by approximately 10-fold in liquid mating (Figure 2C,E). These results indicated that the nature of the donor and recipient is important for plasmid transfer.

The structural integrity of bacteria, especially LPS, is also important for plasmid transfer [37]. For example, Ishiwa [38] revealed that PilV adhesin located at the top of thin pili determines the specificity of the recipient by recognizing its LPS. Duke [39] reported that the plasmids Flac and R1drd19 are easier to transfer to the S218 wild-type strain than its polysaccharide core-related LPS mutant. To acquire colistin resistance, the major mechanism is to alter the structure of cell surface LPS, which results in interference with the electrostatic binding of colistin [5,11]. Our results demonstrated that colistin resistance, acquired either from the IncI2-type plasmid containing the *mcr-1* gene or chromosomal *mgrB* mutation, reduced the transfer frequency of *mcr-1*-related plasmids and co-existed helper plasmid in vitro (Figure 2C–E and Figure 4B,C). The influence was repeatable with the IncP-type plasmid pMCR1.6_P053 harboring *mcr-1.6*. Although various transfer frequencies occur in different types of plasmids, *mcr-1.6* still displayed a significant inhibitory effect on its backbone (Figure 3A). The altering of the donor strain (colistin^R^) in LPS may interfere with the contact with the recipient strain.

Another factor that affects the plasmid transfer is the conjugation conditions. The stabilization of cell-to-cell contact determines the frequency of plasmid transfer. In liquid mating, the conjugation process benefits from mating-pair stabilization either provided by F-pili or type IVb pili that hold cells together and maintain close contact during plasmid transfer [40]. Filter mating fulfills high cell density and close proximity for donor and recipient cells, facilitating the formation of mating pairs for plasmid transfer [30,41,42]. Kosuke reported that the transfer frequency of pCAR1 or pDK1 was significantly different between liquid mating and filter mating [43]. Our results were consistent with this observation (Figure 1).

Environmental factors, like antibiotics, temperature and chemical compounds, are thought to be involved in mediating the plasmid transfer. Hastings [40] reported that ROS response induced by several antibiotics can promote genetic change and the evolution of antibiotic resistance. Aviv [41] reported that the conjugation frequency of pESI was significantly higher at 37 °C than at 27 °C. Besides, the transfer frequency gradually increased with the increasing salt concentration in the conjugation mixture. Additionally, plasmid transfer can be influenced by intestinal tract factors [42,43]. Garcia and Aviv [41,44] reported that the transfer of pES1 and pSLT plasmids were affected by lower oxygen level and bile salt. In this study, we observed that the intestinal environment plays an important role in spreading colistin resistance among bacteria by conjugation (Figure 5A). We observed that additional mucin is ineffective in the conjugation (Figure 5B). Mucin is reported as the barrier in the intestinal epithelium, which is associated with bacterial colonization [31,32], and this barrier may interfere with mating-pair formation. Meanwhile, bile salt and anaerobic conditions facilitated the transfer of the plasmid and, in turn, colistin resistance. The conjugation process is dependent on the cell membrane, as increasing the cell membrane permeability will promote HGT. Xiao found that exposure to a subinhibitory concentration of colistin resulted in a break in the membrane barrier and significantly stimulated the conjugation frequency of *mcr-1*- and bla_NDM-5_-positive [45]. As a digestive secretion, bile can destabilize membranes and disrupt bacterial cellular homeostasis via its detergent-like properties [46] and, presumably, may help promote plasmid transfer. In addition, bile salt has been reported to activate virulence gene expression of *Vibrio cholerae* in anaerobic conditions such as in the small intestine [47]. We suspected that bile salt and limited oxygen concentration might participate in the spread of colistin resistance in vivo. Further studies are required to understand the underlying mechanism.

Polymyxin drugs used in clinics and colistin-containing feeds in animal husbandry could accelerate the spread of colistin resistance. After the discovery of plasmid-mediated mobile colistin resistance in China in 2015, several variants of *mcr* genes have been isolated from fecal samples of humans and livestock [48]. For example, up to 31% of *mcr*-resistant strains were isolated directly from the feces of patients [49]. *mcr-1* [50], *mcr-1.1* [51], *mcr-1.4* [51], *mcr-5* [52], *mcr-10* [53] were isolated from hospital wastewater. Additionally, *mcr-1* [54], *mcr-5.3* [55], and *mcr-8* [56] were isolated from animal waste. Moreover, the presence of *mcr* has been reported in cases from farms, slaughterhouses, and municipal sewage, where human beings and animals live [57,58,59]. Here, we reported that the intestinal tract may be an ideal niche for the plasmid-mediated spread of colistin resistance (and potentially resistance to other antibiotics) among bacteria. This also warns us of the danger of antibiotics overuse.

## 4. Materials and Methods

### 4.1. Bacteria and Growth Condition

The strains and plasmids used in this study are listed in Table 1. All bacteria strains were propagated by Luria-Bertani (LB) broth with the appropriate antibiotic at 37 °C unless otherwise stated.

### 4.2. Growth Curve Assay

Saturated cultures of *K. pneumoniae* A2312NM harboring different plasmids were washed with fresh LB, then diluted 1:100 into 3 mL of LB medium containing the appropriate antibiotic at 37 °C. OD_600_ was measured at a dedicated time.

### 4.3. Competition Assay

A2312NM with the *mcr-1* plasmid or Δ*mcr-1::Km^R^* plasmid were cultured overnight at 37 °C. Saturated cultures were equally inoculated in fresh LB at 37 °C and transferred to LB medium every day at 1:100. Daily samples were counted for viable bacteria on plates with appropriate antibiotics.

### 4.4. Conjugation In Vitro

The liquid conjugation in vitro was performed with modifications [64]. Briefly, log-phase cultures were concentrated 10-fold in LB medium. Equal volumes (100 µL) of donor and recipient strain were mixed and concentrated 5-fold with LB medium containing different concentration of bile salt (0, 0.5%, 1%) or mucin (0, 0.01%, 0.1%). Similarly, treated non-mixed donors or recipients were used as controls. Conjugation proceeded aerobically (standing) or anaerobically (chamber, standing) for 4 h at 37 °C. Samples were serially diluted and plated on selected plates containing the appropriate antibiotics (gentamicin 20 µg/mL, colistin 5 µg/mL, kanamycin 100 µg/mL, chloramphenicol 20 µg/mL, streptomycin 100 µg/mL, apramycin 50 µg/mL, nalidixic acid 10 µg/mL). Representative colonies of conjugants harboring different *mcr-1*-related plasmid were confirmed using PCR to amplify three evenly distributed genes on pHNSHP45 (*mcr-1* primer F-5′TTGCCGTAATTATCCCACCG3′/R-5′TGGAGTGTGCGGTGGGTTTG3′; *parA* primer F-5′GCTGTGTCTGCATTGGTTTG3′/R-5′AGCTACGGGCGCAACAACAC3′; *virB* primer F-5′CCAGACGCAAAGATTGATGG3′/R-5′ATCTGCCAGAAGGACTAAGC3′) and antimicrobial susceptibility testing. Conjugation frequency was calculated as a ratio of the total number of transconjugants to the total number of recipient cells, and spontaneous mutation rates were calculated for the same treated recipient bacteria. For filter mating, the only difference is that the mixed culture is placed on a filter for conjugation.

### 4.5. Conjugation Frequency In Vivo

A streptomycin-pretreated adult mice model was performed as previously described [65] with some modifications. Briefly, a five-week-old CD-1 mouse was provided with drinking water supplied with 5 g/L of streptomycin and 0.05 g/L of aspartame throughout the experiment. Approximately 10^8^ cells of the strains *K. pneumoniae* A2312NM harboring the *mcr-1* plasmid, A2312NM containing the Δ*mcr-1::Km^R^* plasmid, and recipient strain MP13, were mixed equally and intragastrical administered to mice following the treatment with 50 μL of 10% (wt/vol) NaHCO_3_. Fecal samples were collected and homogenized, serially diluted, and then plated on LB medium with the appropriate antibiotics. Conjugants were confirmed using PCR (to amplify *mcr-1*, *parA* and *virB*) and antimicrobial susceptibility testing. The plasmid transfer frequency was determined as a ratio of the total number of transconjugants to the total number of recipient cells.

## Figures and Tables

**Table 1:** Strains and plasmids used in this study.

Strain and Plasmid	Description	Reference or Source
**Strains**		
***E. coli***		
MG1655	Str^R^	[60]
MP13	Gen^R^, Chl^R^, Tc^R^	[61]
Nissle 1917	Nal^R^, Str^R^	[62]
***K. pneumoniae***		
A2312NM	Clinical isolate, Str^R^, Tc^R^	[15]
D20-2	Clinical isolate, Gen^R^	This study
A1502	Clinical isolate, Gen^R^	This study
**Plasmids**		
pHNSHP45	IncI2 type plasmid, harboring *mcr-1* gene, colistin^R^	[8]
pAC22	*mcr-1* gene in pHNSHP45 is replaced by kanamycin resistance, Km^R^	[15]
pAC23	Recombine *mcr-1* gene on pAC22, colistin^R^	[15]
pAC24	*mcr-1* gene in pHNSHP45 is replaced by apramycin resistance*,* Apra^R^	This study
pMCR1.6_P053	IncP type plasmid, harboring mcr-1.6, colistin^R^	[23]
pRK2013	Auxiliary plasmid for mating, Km^R^	[63]

## Data Availability

Not applicable.

## Supplementary Materials

The following supporting information can be downloaded at: https://www.mdpi.com/article/10.3390/antibiotics11070875/s1, Figure S1: Confirmation of the presence of plasmids.
