# Supplementary material for: mcr-1-Mediated In Vitro Inhibition of Plasmid Transfer Is Reversed by the Intestinal Environment
Source: Antibiotics (Basel). 2022 Jun 29;11(7):875. doi: 10.3390/antibiotics11070875 (PMC9311533; doi:10.3390/antibiotics11070875)
Supplement: Supplementary file 1 [file antibiotics-11-00875-s001.zip › antibiotics-1751153-supplementary.pdf]

Supplementary Materials

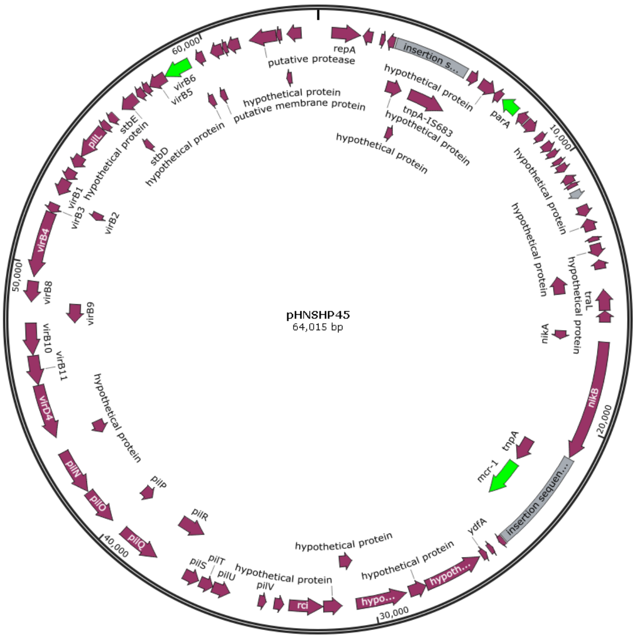

A.

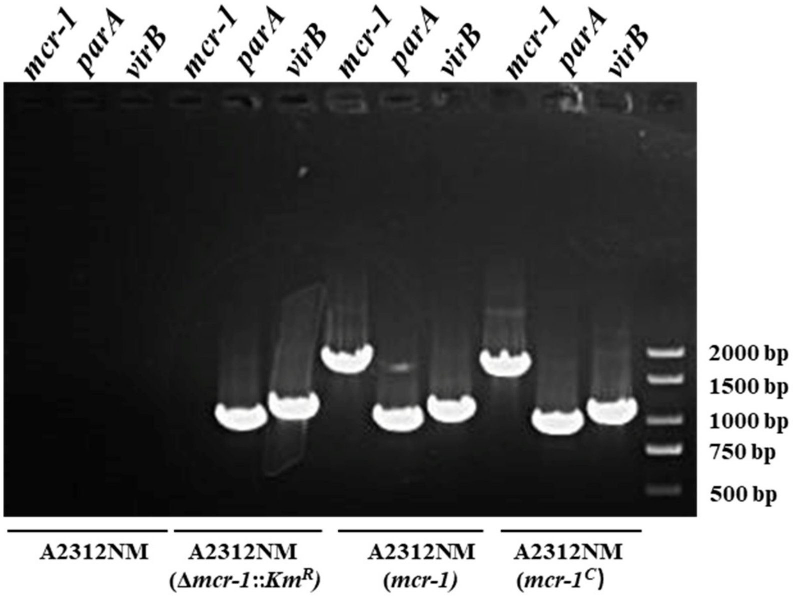

B.

Figure S1. *Confirmation of the presence of plasmids.* (A) Three representative genes *mcr-1*, *parA* and *virB*, were selected on pHNSHP45 evenly (labeled as green). (B) Agarose gel electrophoresis results of A2312NM harboring *mcr-1* plasmid, Δ*mcr-1::Km*^{R} plasmid, or *mcr-1*^{C} plasmid.
